# Brain biopsy in AIDS patients: diagnostic yield and treatment applications

**DOI:** 10.1186/1742-6405-11-4

**Published:** 2014-01-21

**Authors:** Zion Zibly, Itzchak Levy, Vlady Litchevski, Dvora Nass, Chen Hofmann, Jacob Barham, Christian A Graves, Roberto Spiegelmann, Moshe Hadani, Zvi R Cohen

**Affiliations:** 1 Department of Neurosurgery, Sheba Medical Center, Ramat Gan, Israel; 2 Institute of Pathology, Sheba Medical Center, Ramat Gan, Israel; 3 AIDS Center, Sheba Medical Center, Ramat Gan, Israel; 4 Institute of Radiology, Sheba Medical Center, Ramat Gan, Israel; 5 The Oncology Center, Sheba Medical Center, Israel, Ramat Gan, Israel; 6 Department of Pathology, Microbiology, and Immunology, University of South Carolina School of Medicine, Carolina, SC, Columbia; 7 National Institute of Neurological Disorders and Stroke, National Institutes of Health, Bethesda, MD, USA

**Keywords:** HIV, Brain biopsy, CNS AIDS, Neuroimmunology

## Abstract

**Objective:**

Central nervous system involvement in AIDS patients can present at any stage of the disease. Brain lesions detected in imaging studies are usually treated empirically. A brain biopsy is indicated in the absence of clinical and radiologic improvement. In the present study, 16 AIDS patients underwent brain biopsy. We evaluated the diagnostic yield of the brain biopsy and the changes in the disease course.

**Materials and methods:**

Sixteen consecutive AIDS patients (12 men, 4 women; mean age 40.8 years) underwent a brain biopsy at Sheba Medical Center between 1997 and 2009. A retrospective analysis was performed and the clinical outcome was recorded.

**Results:**

Median CD4 count before biopsy was 62.6. Magnetic resonance images revealed multiple lesions in 12 patients and enhancing lesions in 12 patients. A total of 19 biopsies were performed in 16 patients. In the present series, the initial procedures provided a diagnostic yield of 81.25% (13 diagnostic cases from 16 procedures in 16 patients). Two of these patients underwent repeated biopsies that were eventually diagnostic . If repeated biopsies were taken into consideration, the diagnostic yield was 93.75% (15 diagnostic cases in 16 patients). The rate of hemorrhagic complications was 10.5% (2 hemorrhages in 19 procedures).

Pathologic examination revealed parasitic and fungal infections in 6 patients (6/16; 38%), progressive multifocal leukoencephalopathy in 4 patients (4/16; 25%), AIDS encephalopathy in 4 patients (4/16; 25%), and lymphoma in 1 patient (1/16; 6%). One patient had a nonspecific inflammatory process (6%). The treatment modality was modified in 12 patients and led to clinical and radiologic improvement in 8 patients.

**Conclusions:**

Brain biopsy should be considered when empiric treatment of central nervous system lesions in AIDS patients fails. Biopsy is diagnostic in the majority of patients. The diagnosis allows for treatment modifications, which lead to clinical and radiologic improvement in some patients.

## Introduction

Central nervous system (CNS) involvement in AIDS patients is a well-established phenomenon that occurs in approximately 40% to 60% of patients at some stage of the disease. In 10% to 20% of patients, neurologic complications are the first manifestation of HIV infection [1]. The causes of brain involvement include infection, inflammatory changes, and neoplasia [2]. The most common brain lesion in HIV patients worldwide is toxoplasmosis [3], but there is an increased incidence of HIV-associated tuberculosis in focal brain lesions in developing countries [4]. Imaging characteristics of brain lesions in HIV patients have been described [5,6]. The diagnostic yield of brain biopsy in HIV patients has also been evaluated [7-9]. In recent years, due to the availability of advanced multidrug therapies such as highly active antiretroviral therapy (HAART), the disease has gained more chronic characteristics and patient prognosis has greatly improved. The increased longevity of the clinical course of AIDS patients requires the establishment of more aggressive therapeutic approaches in patients with CNS involvement. Brain lesions detected on imaging studies are treated empirically according to current clinical guidelines. Brain biopsy is indicated in the absence of clinical and radiologic improvement, based on the recommendations of the National Institutes of Health (NIH), the Centers for Disease Control and Prevention (CDC), and the HIV Medicine Association of the Infectious Diseases Society of America. The objective of this retrospective study was to analyze the data of 16 AIDS patients who underwent a brain biopsy at Sheba Medical Center in Israel to evaluate the diagnostic yield and changes in the disease course.

## Material and methods

### Patient selection and study approval

The study was conducted according to procedures approved by the Sheba Medical Center Institutional Review Board.

#### Clinical data

Sixteen consecutive AIDS patients underwent a brain biopsy at Sheba Medical Center between 1997 and 2009. Patient characteristics; presenting symptoms; neurologic status; CD4 count; radiologic imaging results, including number of lesions, location of the targeted lesion, and presence of post-operative hemorrhage on computed tomography (CT) scans; and pathologic results were retrospectively analyzed. Treatment changes, and clinical and radiologic outcome were recorded.

#### Surgical procedure

Stereotactic brain biopsies were performed using a Radionics® CRW™ frame and localizer (except for one patient that underwent an open image-guided navigation system biopsy using pituitary forceps). After performing the CT scan and establishing the target (based on fusion with preoperative MR images and performed by the neurosurgeon), the patient was taken to the operating room and serial stereotactic biopsies were obtained under local anesthesia along the established trajectory using a side-cutting biopsy needle. CT scans were obtained up to 2 hours post-procedure and the patients remained in the neurosurgical department for overnight observation.

#### Tissue analysis

Tissue specimens collected in the operation room were placed in individual vials for pathologic analysis; incubated in aerobic, anaerobic, mycobacterium, and fungal cultures; and subjected to PCR analysis for the JC and BK polyomaviruses.

## Results

Twelve patients were men. Mean age at onset of the neurologic manifestations was 40.8 years. The clinical manifestations of the disease were mainly cognitive changes and motor deficits. Mean CD4 count before the brain biopsy was 62.6. A total of 19 brain biopsies were performed in 16 patients. In the present series, the initial procedures provided a diagnostic yield of 81.25% (13 diagnostic cases from 16 procedures in 16 patients). Two of these patients underwent repeated biopsies that were eventually diagnostic. If repeated biopsies were taken into consideration, the diagnostic yield was 93.75% (15 diagnostic cases in 16 patients).

Among the three patients with an initially nondiagnostic biopsy, the initial biopsy revealed a nonspecific inflammatory process in two patients, and the procedure was aborted due to hemorrhage in one patient. Two of these patients underwent a repeated biopsy that was eventually diagnostic.

One of the patients initially diagnosed with a nonspecific inflammatory process underwent two more biopsies and the third biopsy was eventually diagnostic. The second patient initially diagnosed with a nonspecific inflammatory process improved with HAART and did not require a second biopsy.

In the present series, the rate of hemorrhagic complications was 10.5% (2 hemorrhages in 19 procedures). Two of our patients had a hemorrhage; the first had a non-surgical bleeding however we had to abort the procedure and the patient underwent later a second procedure with a diagnostic biopsy. The second patient had a diagnostic biopsy, but due to clinical deterioration underwent evacuation of a hematoma. This patient recovered from the procedure, but did not improve with HAART.

Pathologic examination revealed parasitic and fungal infections in 6 patients (6/16) [38%]: toxoplasmosis, N = 4 [67%], aspergillosis N = 1 [17%], and cryptococcosis N = 1 [17%]), progressive multifocal leukoencephalopathy (PML) in 4 (25%) patients, AIDS encephalopathy in 4 patients (25%), and lymphoma in 1 (6%) patient. One patient had a nonspecific inflammatory process (6%), The treatment modality was modified in 12 patients and led to clinical and radiologic improvement in 8 patients (Table 1).

**Table 1:** Patients demographic and clinical data

	**Diagnosis**	**Age**	**SBX VS open**	**Sex**	**Solitary vs multiple**	**Target location**	**Side**	**Enhancement**	**Edema**	**Presenting symptoms**	**CD4 pre biopsy**	**Treatment**	**Complications**	**Clinical improvement**
1	PML	37	sbx	Male	Multiple	BG	Right	No	None	Combined	69	AZT, 3TC	No	No
2	Non diagnostic × 1,Toxoplasmosis 2nd	36	sbx	Male	Solitary	Frontal	Right	Yes	Yes	Motor	50	AZT, 3TC, Antitoxoplasmosis	Bleeding	No
3	PML	41	sbx	Male	Solitary	BG	Left	No	None	Cognitive	10	AZT, 3TC	No	No
4	Lymphoma	39	sbx	Male	Solitary	BG	Right	Yes	yes	Motor	13	Radiation Therapy	No	Yes
5	PML	32	sbx	Male	Multiple	Frontal	Right	No	None	Combined	57	HAART	No	Yes
6	Toxoplasmosis	24	sbx	Male	Multiple	BG	Left	Yes	Yes	Combined	38	HAART, Antitoxoplasmosis	No	No
7	Toxoplasmosis	29	sbx	Male	Solitary	Frontal	Right	Yes	Yes	Motor	185	HAART, Antitoxoplasmosis	No	Yes
8	Non Diagnostic × 2 Aspergilosis 3rd	78	sbx	Male	Multiple	Parietal occipital frontal	Right	Yes	Yes	Motor	50	HAART, Amphotericin	No	No
9	HIV encephalopathy	33	sbx	Male	Multiple	Occipital	Right	Yes	Yes	Combined	3	HAART	No	Yes
10	PML	48	sbx	Male	Multiple	Frontal	Right	Yes	None	Combined	25	HAART	No	Yes
11	Non diagnostic inflamatory reactive process	41	sbx	Female	Multiple	BG	Left	Yes	Yes	Motor	22	HAART	No	Yes
12	Viral infection	37	sbx	Male	Multiple	Frontal	Left	Yes	Yes	Combined	36	HAART	No	No
13	Cryptoccocal	55	sbx	male	Multiple	Temporal	Left	Yes	Yes	Motor	60	HAART, Amphotericin, Intraconazol	No	No
14	Viral infection	46	sbx	Female	Multiple	Occipital	Right	No	None	Combined	338	HAART	No	Yes
15	Viral infection	46	sbx	Female	Multiple	BG	Right	Yes	Yes	Combined	33	HAART	Bleeding	No
16	Toxoplasmosis	32	open	Female	Multiple	Parietal	Right	Yes	Yes	Combined	14	HAART, Antitoxoplasmosis	No	Yes

## Discussion

Treatment with highly active antiviral drugs (HAART) has resulted in an overall decline in morbidity and mortality among HIV-infected patients with advanced disease [10]. The widespread use of prophylactic regimens has changed the disease pattern and decreased the incidence of opportunistic infections [11]. Vago et al. [12] reported on a large autopsy series that showed a significant reduction in the frequency of HIV-related central nervous system lesions in AIDS patients since the beginning of the HAART era.

In patients with HIV, *Toxoplasma gondii* is the most frequent infectious cause of focal brain lesions [3]. Toxoplasmic encephalitis has a subacute onset with focal neurologic abnormalities frequently accompanied by headache, altered mental status, and fever [13].

Diffuse toxoplasmosis encephalitis should be considered in patients with anti-*T gondii* immunoglobulin G (IgG) antibodies and CD4 T-cell counts of <100/μL who present with unexplained neurologic disease.

Since the introduction of HAART, the incidence of toxoplasmic encephalitis has remained stable or decreased, CNS lymphoma has dramatically declined, PML has slightly increased, [11] and the incidence of HIV encephalopathy has increased [14]. Although combination therapies have decreased overall mortality and the prevalence of CNS opportunistic infections, these therapies may be less effective for preventing the direct effects of HIV-1 on the brain. One of our patients presented with histologic features of AIDS encephalopathy. The majority of patients in our cohort were men with a median age of 40, consistent with other studies [15,16]. These findings are compatible with our data , which is reported to be increasing in prevalence in the HAART era [17].

Thallium SPECT and PET scanning in the diagnosis of lymphoma and PCR analysis of the cerebrospinal fluid for the diagnosis of toxoplasmosis and JC virus have led to a shift in the use invasive diagnostic techniques (brain biopsy) to noninvasive diagnostic methods [5]. The patient in our series that was diagnosed with lymphoma had a solitary enhancing lesion. Although CNS lymphoma generally tends to enhance, atypical characteristics may be observed on MR images of immunocompromised patients [18]. Toxoplasma and lymphoma may have similar appearance on MR imaging. Erdag et al. [18] noted that lymphoma usually appears as a solitary lesion, unlike toxoplasmosis, which appears as a multifocal lesion. Only half of our patients with toxoplasmosis, however, had multifocal disease, similar to a previous report [15].

The majority of patients in our cohort that were diagnosed with PML had nonenhancing lesions, consistent with previous studies [18,19]. Other studies, however, report enhancement on MR images of PML patients that may reach 42% [15], and the development of a mass effect and temporary enhancement on MR images in the early phase of treatment might represent positive predictive factors for prolonged survival [19].

A literature review revealed several descriptions of stereotactic brain biopsy in AIDS patients. The majority of these were reports on patients treated in the pre-HAART era [7-9,16,20-26]. In a recent study, Rosenow et al. [15] divided a large cohort of 246 patients that underwent brain biopsy into two diagnostic groups, before and after the HAART era. The authors concluded that the introduction of HAART led to a steep decrease in the number of biopsies performed annually. Biopsy led to an objective and definitive diagnosis in 96% of patients (48/50), in the patients in the HAART era. Definitive diagnoses were obtained for 91.3% of patients (179/196) in the pre-HAART era. The overall diagnostic yield of biopsy was 92.3% of the total patient cohort. Rosenow et al. [15] suggested that AIDS patients with intra-cerebral lesions should undergo toxoplasmosis titers, and those with negative toxoplasmosis titers should undergo early brain biopsy. In our study, the majority of the patients were treated in the HAART era, which may correlate with the relatively small cohort of patients that underwent stereotactic brain biopsy. The diagnostic yield of the biopsy was high, although lower than previously reported [15].

A total of 19 biopsies were performed in 16 patients; one procedure was aborted due to bleeding and three biopsies were nondiagnostic (15%). Significantly, two patients underwent repeated biopsies that were conclusive. Diagnoses were eventually obtained for 93.7% of patients (15/16). Based on the biopsy findings, the treatment modality was modified in 12 patients and led to clinical and radiologic improvement in 8 patients. Modification of the treatment following brain biopsy has also been reported [1,8].

Hemorrhagic complications of biopsy are reported [16,20,23,25]. The incidence of hemorrhage in our small cohort of patients was higher than that reported in a non-HIV group of patients treated in our department [27]. Although the coagulation profile in our patients was within normal limits, there may have been undetectable coagulopathy in this group of patients. Anticoagulopathy regimens have been suggested by others [16] and pre-biopsy correction of thrombopenia less than 100,000/ml is suggested by Rosenow et al. [15]. A larger cohort of patients is required to establish more definitive conclusions.

## Conclusion

We present the results from a single institute HAART era brain biopsy study in a cohort of 16 patients with active AIDS-associated lesions. Our objective diagnostic rates are consistent with those previously reported, allowing for treatment modification and confirming the usefulness of biopsy in these patients.

## Competing interest

The authors declare that they have no competing interests.

## Authors’ contributions

ZZ and ZRC conducted the study, interpreted the data, and edited the manuscript. IL and VL were the primary care physicians of the patients, analyzed and interpreted the data. ZZ, ZRC, MH, and RS performed the surgical procedures or were part of the decision-making process. DN reviewed all the pathology and wrote the reports. CH reviewed all the radiological images. JB treated the oncology patient and interpreted the data. CG reviewed and interpreted the data and edited the manuscript. All authors read and approved the final manuscript.

## References

[B1] Alesch F Armbruster C Budka H Diagnostic value of stereotactic biopsy of cerebral lesions in patients with AIDS Acta Neurochir (Wien) 1995 11 3–4 214 9 8748784 10.1007/BF01417692

[B2] Clifford DB Focal brain lesions in people with HIV Curr Treat Options Neurol 1999 11 2 167 72 10.1007/s11940-999-0016-6 11096706

[B3] Luft BJ Chua A Central nervous system toxoplasmosis in HIV pathogenesis, diagnosis, and therapy Curr Infect Dis Rep 2000 11 4 358 62 10.1007/s11908-000-0016-x 11095878

[B4] Narain JP Raviglione MC Kochi A HIV-associated tuberculosis in developing countries: epidemiology and strategies for prevention Tuber Lung Dis 1992 11 6 311 21 Epub 1992/12/01 10.1016/0962-8479(92)90033-G 1292709

[B5] Chang L Miller BL McBride D Cornford M Oropilla G Buchthal S Brain lesions in patients with AIDS: H-1 MR spectroscopy Radiology 1995 11 2 525 31 7480706 10.1148/radiology.197.2.7480706

[B6] Pomper MG Constantinides CD Barker PB Bizzi A Dobgan AS Yokoi F Quantitative MR spectroscopic imaging of brain lesions in patients with AIDS: correlation with [11C-methyl]thymidine PET and thallium-201 SPECT Acad Radiol 2002 11 4 398 409 10.1016/S1076-6332(03)80185-X 11942654

[B7] Gervasoni C Castagna A Rocca A Vago L Cinque P A comparison of brain biopsy and CSF-PCR in the diagnosis of CNS lesions in AIDS patients J Neurol 1997 11 1 35 9 9007743 10.1007/pl00007727

[B8] Viswanathan R Ironside J Bell JE Brettle RP Whittle IR Stereotaxic brain biopsy in AIDS patients: does it contribute to patient management? Br J Neurosurg 1994 11 3 307 11 10.3109/02688699409029618 7946019

[B9] Zimmer C Marzheuser S Patt S Rolfs A Gottschalk J Weigel K Stereotactic brain biopsy in AIDS J Neurol 1992 11 7 394 400 1328543 10.1007/BF00812158

[B10] Palella FJ Jr Delaney KM Moorman AC Loveless MO Fuhrer J Satten GA Declining morbidity and mortality among patients with advanced human immunodeficiency virus infection. HIV outpatient study investigators N Engl J Med 1998 11 13 853 60 10.1056/NEJM199803263381301 9516219

[B11] Ammassari A Cingolani A Pezzotti P De Luca DA Murri R Giancola ML AIDS-related focal brain lesions in the era of highly active antiretroviral therapy Neurology 2000 11 8 1194 200 11071499 10.1212/wnl.55.8.1194

[B12] Vago L Bonetto S Nebuloni M Duca P Carsana L Zerbi P Pathological findings in the central nervous system of AIDS patients on assumed antiretroviral therapeutic regimens: retrospective study of 1597 autopsies Aids 2002 11 14 1925 8 10.1097/00002030-200209270-00009 12351952

[B13] Renold C Sugar A Chave JP Perrin L Delavelle J Pizzolato G Toxoplasma encephalitis in patients with the acquired immunodeficiency syndrome Med (Baltimore) 1992 11 4 224 39 10.1097/00005792-199207000-00005 1355579

[B14] Neuenburg JK Brodt HR Herndier BG Bickel M Bacchetti P Price RW HIV-related neuropathology, 1985 to 1999: rising prevalence of HIV encephalopathy in the era of highly active antiretroviral therapy J Acquir Immune Defic Syndr 2002 11 2 171 7 10.1097/00126334-200210010-00007 12394795

[B15] Rosenow JM Hirschfeld A Utility of brain biopsy in patients with acquired immunodeficiency syndrome before and after introduction of highly active antiretroviral therapy Neurosurgery 2007 11 1 130 40 discussion 40–1 10.1227/01.neu.0000279733.28768.ff 17621028

[B16] Gildenberg PL Gathe JC Jr Kim JH Stereotactic biopsy of cerebral lesions in AIDS Clin Infect Dis 2000 11 3 491 9 10.1086/313685 10722433

[B17] McArthur JC HIV dementia: an evolving disease J of neuroimmunol 2004 11 1–2 3 10 Epub 2004/12/08 15579274 10.1016/j.jneuroim.2004.08.042

[B18] Erdag N Bhorade RM Alberico RA Yousuf N Patel MR Primary lymphoma of the central nervous system: typical and atypical CT and MR imaging appearances AJR Am J Roentgenol 2001 11 5 1319 26 10.2214/ajr.176.5.1761319 11312202

[B19] Thurnher MM Post MJ Rieger A Kleibl-Popov C Loewe C Schindler E Initial and follow-up MR imaging findings in AIDS-related progressive multifocal leukoencephalopathy treated with highly active antiretroviral therapy AJNR Am J Neuroradiol 2001 11 5 977 84 11337345 PMC8174932

[B20] Gildenberg PL Langford L Kim JH Trujillo R Stereotactic biopsy in cerebral lesions of AIDS Acta Neurochir Suppl (Wien) 1993 11 68 70 8109306 10.1007/978-3-7091-9297-9_15

[B21] Levy RM Breit R Russell E Dal Canto MC MRI-guided stereotaxic brain biopsy in neurologically symptomatic AIDS patients J Acquir Immune Defic Syndr 1991 11 3 254 60 1992103

[B22] Levy RM Russell E Yungbluth M Hidvegi DF Brody BA Dal Canto MC The efficacy of image-guided stereotactic brain biopsy in neurologically symptomatic acquired immunodeficiency syndrome patients Neurosurgery 1992 11 2 186 9 discussion 9–90 10.1227/00006123-199202000-00006 1545885

[B23] Nielsen CJ Gjerris F Pedersen H Jensen FK Wagn P Brain biopsy in AIDS. Diagnostic value and consequence Acta Neurochir (Wien) 1994 11 1–2 99 102 7942191 10.1007/BF01808555

[B24] O'Doherty MJ Lewis P Nunan TO Brain biopsy for intracranial mass lesions in AIDS Lancet 1993 11 8839 242 3 10.1016/0140-6736(93)90106-Q 8093524

[B25] Pell MF Thomas DG Whittle IR Stereotactic biopsy of cerebral lesions in patients with AIDS Br J Neurosurg 1991 11 6 585 9 10.3109/02688699109002881 1772604

[B26] Preuss M Schumacher HC Bergs C Brock M Brain biopsy for intracranial mass lesions in AIDS Lancet 1993 11 8839 242 3 10.1016/0140-6736(93)90106-Q 8093525

[B27] Grossman R Sadetzki S Spiegelmann R Ram Z Haemorrhagic complications and the incidence of asymptomatic bleeding associated with stereotactic brain biopsies Acta Neurochir (Wien) 2005 11 6 627 31 discussion 31 10.1007/s00701-005-0495-5 15821863

